# Turnover Among Early-Career Advanced Practice Clinicians

**DOI:** 10.1001/jamanetworkopen.2025.8638

**Published:** 2025-05-05

**Authors:** Max J. Hyman, Kim Litwack, Susanne A. Quallich, Andrew W. Schram, Ted A. Skolarus, Parth K. Modi

**Affiliations:** 1 The Center for Health and the Social Sciences, The University of Chicago, Chicago, Illinois; 2 College of Health Professions and Sciences, University of Wisconsin–Milwaukee, Milwaukee; 3 Department of Urology, University of Michigan, Ann Arbor; 4 Department of Medicine, The University of Chicago, Chicago, Illinois; 5 Department of Surgery, The University of Chicago, Chicago, Illinois

## Abstract

This cohort study examines the frequency of moves to other practices among advanced practice clinicians (APCs) to understand rates of turnover among early-career APCs, which has implications for their hiring, training, and retention.

## Introduction

Advanced practice clinicians (APCs) will join the US health care workforce in 5-fold greater numbers than physicians from 2023 to 2033.^1^ Although a growing literature investigates the quality and cost-effectiveness of APCs, job turnover among APCs remains underexamined. Turnover in health care has significant costs,^2^ and because of fewer formal subspecialty training or certification requirements, APCs have lower barriers to job and specialty changes than physicians.^3^ As the employment rate for APCs increases, understanding their job turnover is valuable in hiring, training, and retention decisions.

## Methods

This retrospective cohort study used Medicare Data on Provider Practice and Specialty files to identify APCs who first billed Medicare Part B between 2010 and 2021.^4^ These data include any clinician who submitted professional claims. The University of Chicago institutional review board approved this study and waived the need for informed consent because the data were deidentified. We adhered to the STROBE reporting guideline. Statistical analysis was performed from March 4 to November 25, 2024, using SAS software, version 9.4.

We identified the first month of the earliest year in which a nurse practitioner, physician assistant, certified registered nurse anesthetist, certified nurse midwife, or clinical nurse specialist billed, requiring no billing in at least the 2 previous years. Each month thereafter, we determined whether an APC billed under a new practice, in which practice affiliation was defined by the tax identification number (TIN). Stoppage of billing under the original TIN followed by billing under the new TIN for at least 2 consecutive months constituted practice movement. As practice movement may represent consolidation events (eg, merger), we excluded moves if a physician changed TINs concurrent with the APC (8.2% of movement events were excluded due to reclassification as consolidation events).

We conducted a time-to-event analysis of the number of months from first to last billing in the first practice. Practice specialty was characterized by the physician specialty that comprised greater than 50% of physicians in the practice and otherwise was considered a multispecialty group (eTable in Supplement 1).

## Results

The total sample size of all APCs was 217 487 (nurse practitioners, 132 670; physician assistants, 62 491; certified registered nurse anesthetists, 15 934; certified nurse midwives, 5066; clinical nurse specialists, 1236). Of 217 487 APCs (mean [SD] age, 37.1 [10.0] years; 180 442 women [83.0%]) who first billed between 2010 and 2021, 58 337 (26.8%) moved practices (median time to event, 13.0 [IQR, 6.0-26.0] months), and 159 150 (73.2%) did not and were right censored (median time to event, 27.0 [IQR, 11.0-55.0] months). Greater proportions of certified registered nurse anesthetists (34.9% [n = 5560]) and physician assistants (30.7% [n = 19 164]) moved practices than nurse practitioners (24.6% [n = 32 699]). Male APCs were more likely to move than female APCs (29.9% [11 084 of 37 045] vs 26.2% [47 253 of 180 442]). APCs who moved were in smaller practices than those who did not move (median number of physicians, 16 [IQR, 3-88] vs 57 [IQR, 7-335]; median number of other APCs, 10 [IQR, 2-43] vs 29 [IQR, 5-126]).

Cumulatively, 14.4% of APCs moved practices within 1 year of first billing and 29.8% within 3 years (Table). Movement was most common in hospital-based specialties (20.7% and 43.0% moved within 1 and 3 years, respectively) and least common in medical subspecialties (14.4% and 30.2%) and obstetrics and gynecology (12.1% and 23.3%) (Figure).

**Table.** Cumulative Percentage of APCs Who Moved Practices Within 1, 2, 3, and 5 Years of First Billing Medicare

Type of APC	No.	Cumulative % (95% CI) of APCs who moved			
		1 y	2 y	3 y	5 y
All	217 487	14.4 (14.2-14.6)	23.7 (23.5-23.9)	29.8 (29.6-30.1)	36.9 (36.7-37.2)
Nurse practitioners	132 670	14.4 (14.2-14.6)	23.0 (22.8-23.3)	28.4 (28.1-28.7)	34.6 (34.3-35.0)
Physician assistants	62 491	15.4 (15.1-15.7)	25.9 (25.5-26.3)	33.1 (32.7-33.6)	40.9 (40.4-41.4)
Certified registered nurse anesthetists	15 934	13.1 (12.5-13.6)	24.2 (23.5-24.9)	32.7 (31.9-33.5)	43.2 (42.2-44.1)
Certified nurse midwives	5066	8.0 (7.2-8.8)	12.2 (11.2-13.3)	15.5 (14.3-16.8)	19.7 (18.3-21.3)
Clinical nurse specialists	1236	8.3 (6.9-10.1)	14.9 (12.9-17.2)	18.3 (16.1-20.9)	22.1 (19.5-25.0)

Abbreviation: APC, advanced practice clinician.

**Figure.**
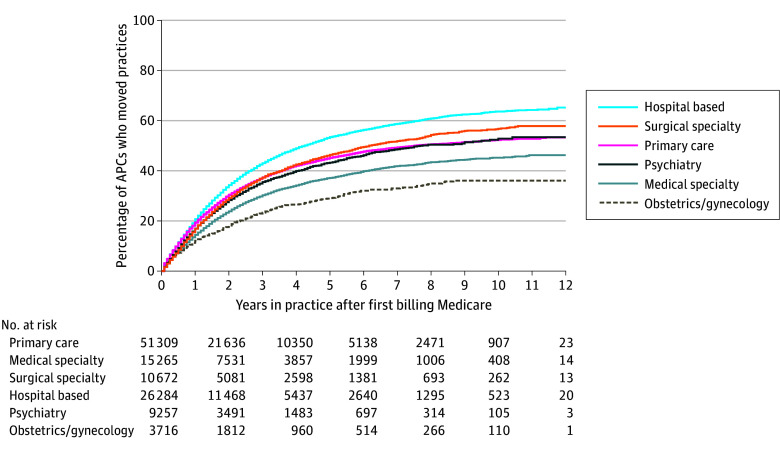
Number of Years Until Advanced Practice Clinicians (APCs) Moved Practices After First Billing Medicare by Broad Practice Specialty This analysis excludes APCs who first billed Medicare in a multispecialty group (N = 100 984). The specialties included in each broad specialty are described in the eTable in Supplement 1.

## Discussion

Approximately 30% of APCs who first billed Medicare between 2010 and 2021 moved practices within 3 years. Movement varied by sex, licensure type, practice size, and practice specialty. The high turnover rate we observed has significant implications for hiring and training investments made by APCs and medical practices. Further work should investigate practice characteristics, specific tasks, remuneration, and other potential factors associated with practice turnover.

This study must be considered amid its limitations. These data are limited to APCs who independently bill Medicare. The first billing under a TIN may not correspond to the start of employment (eg, onboarding before independent billing). Movement within a TIN (eg, changing specialties within a large multispecialty group) was not captured. Nevertheless, these results help us better understand the frequency of practice movement for early-career APCs, which has implications for their hiring, training, and retention.

## Notes

Supplement 1. **eTable.** Practice Specialty and Type Groupings of Medicare Data on Provider Practice and Specialty (MD-PPAS) Specialties

Supplement 2.
Data Sharing Statement
