# Errors in Methods and Results

**DOI:** 10.1001/jamanetworkopen.2025.21128

**Published:** 2025-06-10

In the Research Letter titled, “Turnover Among Early-Career Advanced Practice Clinicians”^1^ published May 5, 2025, there were 2 minor typographical errors in the Methods and data errors in the Results that incorrectly reported the numbers of advanced practice clinicians (APCs). The numbers of APCs in the first paragraph of the Results have been corrected to indicate that there were 5560 certified registered nurse anesthetists, 19 164 physician assistants, 32 699 nurse practitioners, 11 084 male APCs, and 47 253 female APCs who moved practices. The numbers of APCs in the second paragraph have been removed because they did not accurately reflect the cumulative rate of movement between practices. This article has been corrected.^1^
