# Supplementary material for: Turnover Among Early-Career Advanced Practice Clinicians
Source: JAMA Netw Open. 2025 May 5;8(5):e258638. doi: 10.1001/jamanetworkopen.2025.8638 (PMC12053513; doi:10.1001/jamanetworkopen.2025.8638)
Supplement: Supplement 1. — eTable. Practice Specialty and Type Groupings of Medicare Data on Provider Practice and Specialty (MD-PPAS) Specialties [file jamanetwopen-e258638-s001.pdf]

Supplemental Online Content

Hyman MJ, Litwack K, Quallich SA, Schram AW, Skolarus TA, Modi PK. Turnover among early-career advanced practice clinicians. *JAMA Netw Open*. 2025;8(5):e258638. doi:10.1001/jamanetworkopen.2025.8638

eTable. Practice Specialty and Type Groupings of Medicare Data on Provider Practice and Specialty (MD-PPAS) Specialties

This supplemental material has been provided by the authors to give readers additional information about their work.

© 2025 Hyman MJ et al. *JAMA Network Open.*

eTable. Practice Specialty and Type Groupings of Medicare Data on Provider Practice and Specialty (MD-PPAS) Specialties

MD-PPAS Specialty	Broad Practice Specialty
Family practice	Primary Care
General practice	Primary Care
Internal medicine	Primary Care
Osteopathic manipulative medicine	Primary Care
Preventative medicine	Primary Care
Undersea and hyperbaric medicine	Primary Care
Geriatric medicine	Primary Care
Hospice and palliative care	Primary Care
Addiction medicine	Medical Specialty
Allergy/Immunology	Medical Specialty
Adult congenital heart disease	Medical Specialty
Advanced heart failure and transplant cardiology	Medical Specialty
Cardiac electrophysiology	Medical Specialty
Cardiovascular disease (cardiology)	Medical Specialty
Interventional cardiology	Medical Specialty
Dermatology	Medical Specialty
Micrographic dermatologic surgery	Medical Specialty
Endocrinology	Medical Specialty
Gastroenterology	Medical Specialty
Hematology	Medical Specialty
Hematology/Oncology	Medical Specialty
Hematopoietic cell transplantation and cellular therapy	Medical Specialty
Medical oncology	Medical Specialty
Infectious disease	Medical Specialty
Medical genetics and genomics	Medical Specialty
Nephrology	Medical Specialty
Neurology	Medical Specialty
Pulmonary disease	Medical Specialty
Sleep medicine	Medical Specialty
Rheumatology	Medical Specialty
Medical toxicology	Medical Specialty
Cardiac surgery	Surgical Specialty
Colorectal surgery	Surgical Specialty
General surgery	Surgical Specialty

© 2025 Hyman MJ et al. *JAMA Network Open.*

Neurosurgery	Surgical Specialty
Ophthalmology	Surgical Specialty
Orthopedic Surgery	Surgical Specialty
Otolaryngology	Surgical Specialty
Hand surgery	Surgical Specialty
Plastic and reconstructive surgery	Surgical Specialty
Surgical oncology	Surgical Specialty
Thoracic surgery	Surgical Specialty
Urology	Surgical Specialty
Vascular Surgery	Surgical Specialty
Peripheral vascular disease	Surgical Specialty
Anesthesiology	Hospital-Based Specialty
Interventional pain management	Hospital-Based Specialty
Pain management	Hospital-Based Specialty
Critical care (intensivists)	Hospital-Based Specialty
Emergency medicine	Hospital-Based Specialty
Hospitalist	Hospital-Based Specialty
Pathology	Hospital-Based Specialty
Physical medicine and rehabilitation	Hospital-Based Specialty
Sports medicine	Hospital-Based Specialty
Radiation oncology	Hospital-Based Specialty
Diagnostic radiology	Hospital-Based Specialty
Interventional radiology	Hospital-Based Specialty
Nuclear medicine	Hospital-Based Specialty
Gynecological/Oncology	Obstetrics and Gynecology
Obstetrics/Gynecology	Obstetrics and Gynecology
Geriatric psychiatry	Psychiatry
Neuropsychiatry	Psychiatry
Psychiatry	Psychiatry

© 2025 Hyman MJ et al. *JAMA Network Open.*
