# Supplementary material for: Turnover Among Early-Career Advanced Practice Clinicians
Source: JAMA Netw Open. 2025 May 5;8(5):e258638. doi: 10.1001/jamanetworkopen.2025.8638 (PMC12053513; doi:10.1001/jamanetworkopen.2025.8638)
Supplement: Supplement 2. — Data Sharing Statement [file jamanetwopen-e258638-s002.pdf]

Data Sharing Statement

Hyman. Turnover Among Early-Career Advanced Practice Clinicians. *JAMA Netw Open*. Published May 05, 2025. doi:10.1001/jamanetworkopen.2025.8638

Data
Data available: No
